# Regulation of Coronafacoyl Phytotoxin Production by the PAS-LuxR Family Regulator CfaR in the Common Scab Pathogen *Streptomyces scabies*

**DOI:** 10.1371/journal.pone.0122450

**Published:** 2015-03-31

**Authors:** Zhenlong Cheng, Luke Bown, Kapil Tahlan, Dawn R. D. Bignell

**Affiliations:** Department of Biology, Memorial University of Newfoundland, St. John’s, NL A1B 3X9, Canada; Belgian Nuclear Research Centre SCK•CEN, BELGIUM

## Abstract

Potato common scab is an economically important crop disease that is characterized by the formation of superficial, raised or pitted lesions on the potato tuber surface. The most widely distributed causative agent of the disease is *Streptomyces scabies*, which produces the phytotoxic secondary metabolite thaxtomin A that serves as a key virulence factor for the organism. Recently, it was demonstrated that *S*. *scabies* can also produce the phytotoxic secondary metabolite coronafacoyl-L-isoleucine (CFA-L-Ile) as well as other related metabolites in minor amounts. The expression of the biosynthetic genes for CFA-L-Ile production is dependent on a PAS-LuxR family transcriptional regulator, CfaR, which is encoded within the phytotoxin biosynthetic gene cluster in *S*. *scabies*. In this study, we show that CfaR activates coronafacoyl phytotoxin production by binding to a single site located immediately upstream of the putative -35 hexanucleotide box within the promoter region for the biosynthetic genes. The binding activity of CfaR was shown to require both the LuxR and PAS domains, the latter of which is involved in protein homodimer formation. We also show that CFA-L-Ile production is greatly enhanced in *S*. *scabies* by overexpression of both *cfaR* and a downstream co-transcribed gene, *orf1*. Our results provide important insight into the regulation of coronafacoyl phytotoxin production, which is thought to contribute to the virulence phenotype of *S*. *scabies*. Furthermore, we provide evidence that CfaR is a novel member of the PAS-LuxR family of regulators, members of which are widely distributed among actinomycete bacteria.

## Introduction

The genus *Streptomyces* consists of hundreds of species of Gram-positive filamentous actinobacteria that are recognized for their ability to produce a large variety of useful secondary metabolites, including many medically and agriculturally important compounds [1]. In addition, some species are notable for their ability to cause important crop diseases such as potato common scab (CS), which is characterized by the formation of superficial, erumpent (raised) or pitted lesions on the potato tuber surface [2]. Such lesions negatively impact the quality and market value of the potato tubers and cause significant economic losses to potato growers. In Canada, losses associated with CS during the 2002 growing season were estimated at $15.3–17.3 million dollars [3], and in Australia, the disease has been estimated to cause losses of approximately 4% of the total industry value [4]. Furthermore, it has been reported that CS can also decrease the overall yield of the potato crop and increase the number of smaller tubers in the yield [5].

*Streptomyces scabies* (syn. *scabiei*) is the best characterized and most widely distributed *Streptomyces* spp. that causes CS disease [2]. The key virulence factor produced by *S*. *scabies* and other CS-causing pathogens is a phytotoxic secondary metabolite called thaxtomin A, which functions as a cellulose synthesis inhibitor [6–10]. It has been shown by several groups that there is a positive correlation between the pathogenicity of scab-causing organisms and the production of the thaxtomin A phytotoxin [11–15]. Recently, it was demonstrated that *S*. *scabies* strain 87–22 also produces metabolites that are structurally related to the coronatine (COR) phytotoxin, which contributes to the virulence phenotype of the Gram-negative plant pathogen *Pseudomonas syringae* [16]. COR functions in promoting the invasion and multiplication of *P*. *syringae* within the plant host, it contributes to disease symptom development during *P*. *syringae* infection, and it enhances the disease susceptibility of the plant in uninfected regions [17]. In *P*. *syringae*, COR is produced by linking coronafacic acid (CFA) to coronamic acid (CMA), a reaction that is thought to be catalyzed by the coronafacate ligase (Cfl) enzyme [18]. Although *S*. *scabies* lacks the ability to produce COR due to the absence of the CMA biosynthetic genes, it does harbour homologues of genes involved in CFA biosynthesis as well as a *cfl* homologue [19]. Recent work from our laboratory demonstrated that this organism produces the coronafacoyl compound CFA-L-Ile as a major product along with other related molecules in minor amounts [16]. Furthermore, mutational studies in *S*. *scabies* combined with bioactivity studies of the pure CFA-L-Ile molecule support the notion that this molecule functions as a phytotoxin and contributes to the virulence phenotype of *S*. *scabies* [16, 19].

**Fig 1:**
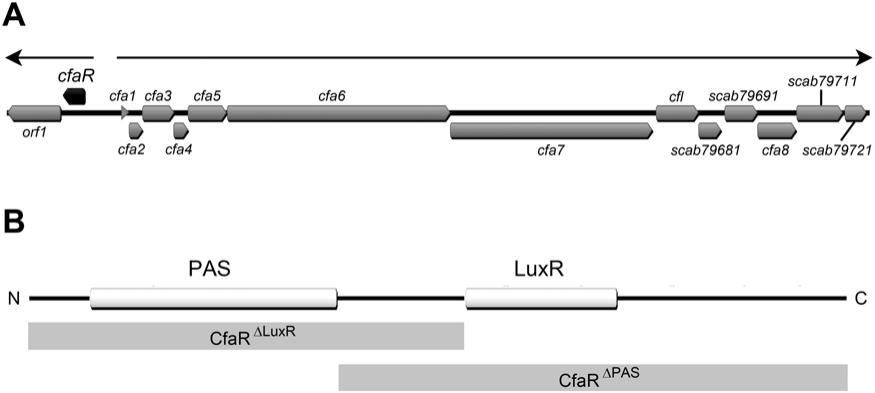
Organization of the *Streptomyces scabies* 87–22 coronafacoyl phytotoxin biosynthetic gene cluster and domain structure of the CfaR protein. (A) The block arrows represent the coding sequences within the gene cluster, and the direction of each arrow indicates the direction of transcription. The *cfaR* gene is indicated in black while all other genes are shown in gray. The thin arrows at the top of the image indicate the two transcription units that have been identified [19]. (B) The CfaR protein consists of an N-terminal PAS sensory domain (PF00989) and a C-terminal LuxR-type DNA binding domain (PF00196). The gray shaded boxes below the image show the domain composition of the truncated forms of CfaR (CfaR^ΔLuxR^ and CfaR^ΔPAS^) that were constructed and used in this study.

The biosynthetic gene cluster for production of the coronafacoyl phytotoxins in *S*. *scabies* is composed of at least 15 genes (Fig 1A), of which 13 are co-transcribed as a single polycistronic mRNA transcript [19]. The remaining two genes are oriented in the opposite direction to the other genes and are co-transcribed as a separate transcript [19]. The first gene in this two-gene operon, *scab79591* (herein referred to as *cfaR*), encodes a 265 amino acid protein belonging to the PAS-LuxR family of transcriptional regulators, which are only found in the actinomycetes. Members of this family contain an N-terminal PAS (PER-ARNT-SIM) domain and a C-terminal LuxR-type domain (Fig 1B), and are often associated with secondary metabolite biosynthetic gene clusters. PAS domains belong to a sensing module superfamily that recognize stimuli such as light, oxygen, redox potential or ligands in order to modulate the regulatory activity of the corresponding protein in which they are present [20]. The LuxR-type domain is named after the *Vibrio fischeri* LuxR protein where the domain was first identified, and it contains a helix-turn-helix (HTH) motif that is typically involved in binding to specific DNA sequences called *lux-*boxes within target promoter(s) for transcription activation [21]. The best characterized PAS-LuxR family member is PimM, which in *Streptomyces natalensis* binds to eight promoters and activates expression of the biosynthetic genes for production of the polyene antifungal antibiotic pimaricin (also known as natamycin) [22, 23]. Other family members that have been described include PteF, which controls the production of filipin in *Streptomyces avermitilis* [24], AURJ3M, which is a positive activator of aureofuscin biosynthesis in *Streptomyces aureofuscus* [25] and SlnM, which activates production of natamycin in *Streptomyces lyticus* [26]. In *S*. *scabies*, the CfaR protein has been shown to function as a transcriptional activator for CFA-L-Ile phytotoxin production [16, 19]; however, there is currently no information as to how the protein regulates phytotoxin production.

In this study, we set out to characterize the mechanism of regulation of coronafacoyl phytotoxin biosynthetic gene expression by the CfaR protein. We show that the protein binds to a single imperfect palindromic sequence located immediately upstream of the putative -35 hexanucleotide box in the *cfa1* promoter region, which drives the expression of the biosynthetic genes for phytotoxin production. We also show that the DNA binding activity of CfaR depends on both the LuxR and PAS domains, and that the PAS domain is required for the formation of CfaR homodimers. Furthermore, we demonstrate that a high level of CFA-L-Ile production occurs in *S*. *scabies* when *cfaR* is overexpressed together with the downstream co-transcribed gene, *orf1*. This, together with phylogenetic analyses of CfaR and other PAS-LuxR proteins, indicates that CfaR is a novel member of the PAS-LuxR family of transcriptional regulators.

## Materials and Methods

### Bacterial strains, cultivation and maintenance

Bacterial strains used in this study are listed in Table 1. *Escherichia coli* strains were routinely cultivated in Luria-Bertani (LB) Lennox medium (Fisher Scientific, Canada) at 37°C unless otherwise stated. Where required, the LB medium was supplemented with kanamycin or apramycin (Sigma Aldrich, Canada) at 50 μg/mL final concentration, or with chloramphenicol (MP Biomedicals North America, USA) at 25 μg/mL final concentration. *E*. *coli* strains were maintained at -80°C in 20% v/v glycerol [27]. *S*. *scabies* strains were routinely cultured at 25°C or 28°C on potato mash agar (PMA; 5% w/v mashed potato flakes, 2% w/v agar) solid medium or in trypticase soy broth (TSB; BD Biosciences, Canada), nutrient broth (BD Biosciences, Canada) and soy flour mannitol broth (SFMB) liquid media [28]. When necessary, the growth medium was supplemented with apramycin or thiostrepton (Sigma Aldrich, Canada) at 50 or 25 μg/mL final concentration, respectively. Seed cultures for RNA extraction were prepared by inoculating 50 μL of a *S*. *scabies* spore stock into 5 mL of TSB followed by incubation for 24–48 hr until dense mycelial growth was obtained. The seed cultures (0.5 mL) were subsequently used to inoculate 25 mL of SFMB in 125 mL flasks, which were incubated at 25°C and 200 rpm for 4 days. Cultures for small scale CFA-L-Ile extraction were prepared by inoculating TSB seed cultures (200 μL) into 5 mL of SFMB in 6 well plates (Fisher Scientific, Canada) and then incubating at 25°C and 125 rpm for 7 days. *S*. *scabies* strains were maintained at -80°C as spore suspensions in 20% v/v glycerol [28].

**Table 1:** Bacterial strains and plasmids used in this study.

**Strain or plasmid**	**Description**	**Resistance** ^†^	**Reference or source**
***Streptomyces scabies* strains**			
87–22	Wild-type strain	n/a	[15]
Δ*txtA*	*S*. *scabies* 87–22 containing a deletion of the *txtA* thaxtomin biosynthetic gene	Apra^R^	[29]
***Escherichia coli* strains**			
DH5α	General cloning host	n/a	Gibco-BRL
NEB 5- α	DH5 α derivative, high efficiency competent cells	n/a	New England Biolabs
BL21(DE3)	Protein expression strain	n/a	New England Biolabs
ET12567/pUZ8002	*dam* ^*–*^, *dcm* ^*–*^, *hsdS* ^*–*^; nonmethylating conjugation host	Kan^R^, Cml^R^	[30]
**Plasmids**			
pET-30b	N- or C- terminal 6 × histidine fusion tag protein expression vector with T7 promoter and *lac* operator	Kan^R^	Novagen
pET-30b/CfaR	pET-30b derivative carrying a DNA fragment for expression of the CfaR^full^–HIS_6_ protein	Kan^R^	This study
pET-30b/CfaR1.1	pET-30b derivative carrying a DNA fragment for expression of the CfaR^ΔLuxR^–HIS_6_ protein	Kan^R^	This study
pET-30b/CfaR1.3	pET-30b derivative carrying a DNA fragment for expression of the CfaR^ΔPAS^–HIS_6_ protein	Kan^R^	This study
pRLDB50-1a	*Streptomyces* expression plasmid; carries the strong, constitutive promoter *ermE*p*and integrates into theφC31 *attB* site	Apra^R^, Thio^R^	[19]
pRLDB51-1	*scab79591* (*cfaR*) overexpression plasmid derived from pRLDB50-1a	Apra^R^, Thio^R^	[19]
pRLDB81	*scab79581* (*orf1*) overexpression plasmid derived from pRLDB50-1a	Apra^R^, Thio^R^	This study
pRLDB891	*cfaR + orf1* overexpression plasmid derived from pRLDB50-1a	Apra^R^, Thio^R^	This study

^†^ Apra^R^, Thio^R^, Kan^R^ and Cml^R^ = apramycin, thiostrepton, kanamycin and chloramphenicol resistance, respectively.

n/a = not applicable.

### Plasmids, primers and DNA manipulation

Plasmids used in this study are listed in Table 1. Plasmids were manipulated in *E*. *coli* using standard procedures [27]. All oligonucleotides used in reverse transcription, PCR, sequencing and electrophoretic mobility shift assays were purchased from Integrated DNA Technologies (USA) and are listed in S1 Table. DNA sequencing was performed by The Centre for Applied Genomics (TCAG; Canada). *Streptomyces* genomic DNA was isolated from mycelia harvested from 2-day old nutrient broth cultures using the DNeasy Blood & Tissue Kit as per the manufacturer’s protocol (QIAgen Inc, Canada).

### Construction of protein expression plasmids

Three forms of the *cfaR* gene, one encoding the full length protein (CfaR^full^), one encoding the first 140 amino acids of the protein with the PAS domain (CfaR^ΔLuxR^), and one encoding the C-terminal 174 amino acids of the protein and harbouring the LuxR domain (CfaR^ΔPAS^), were amplified by PCR using Phusion DNA Polymerase (New England Biolabs, Canada) according to the manufacturer’s instructions, except that DMSO (5% v/v final concentration) was included in the reactions. The resulting products were digested with NdeI and HindIII (New England Biolabs, Canada) and were ligated into similarly digested pET30b to generate the C-terminal 6 × HIS-tagged full length and truncated CfaR expression plasmids. The constructed expression plasmids were sequenced to confirm the fidelity of the inserts, after which they were transformed into *E*. *coli* BL21(DE3) cells using the one step method [31].

### Protein overexpression and purification

For expression of CfaR^full^ –HIS_6_ and CfaR^ΔLuxR^ –HIS_6_, the *E*. *coli* cells were grown at 28°C in 500 mL of LB containing kanamycin until an OD_600_ of 0.6 was reached, after which isopropyl 1-thio-β-D-glucopyranoside (IPTG) was added to a final concentration of 1 mM and the cells were incubated for an additional 4 h. To express CfaR^ΔPAS^–HIS_6_, cells were grown at 25°C to an OD_600_ of 0.6, after which they were induced with IPTG (0.25 mM final concentration) and were incubated for an additional 5 h. The cells were harvested and resuspended in buffer consisting of 20 mM sodium phosphate, 500 mM sodium chloride and 30 mM imidazole (pH 7.4), and were lysed using a French press (SLM Instruments Inc., USA). The soluble proteins were purified using an ÄKTA pure FPLC system with a HiTrap IMAC FF 1 mL column at 4°C according to the manufacturer’s recommendations (GE Healthcare, Canada). The collected fractions were analyzed by SDS-PAGE on a 12% gel, and those fractions containing protein were pooled and desalted by FPLC using a HiTrap Desalting 5 mL column (GE Healthcare, Canada). The protein concentration in each preparation was determined by the Bradford method [32], and the proteins were stored at -80°C in buffer containing 20 mM sodium phosphate, 150 mM NaCl and 20% glycerol (pH = 7.8).

### Total RNA isolation

*S*. *scabies* mycelia from 4-day old SFMB cultures were harvested by centrifugation, and approximately 0.5 g of the cell pellet was placed into a sterile 2 mL microcentrifuge tube. Total RNA was isolated using an innuPREP Bacteria RNA Kit and a SpeedMill PLUS tissue homogenizer (Analytik Jena AG, Germany) as per the manufacturer’s instructions. The resulting RNA samples were treated with DNase I (New England Biolabs, Canada) as directed by the manufacturer to remove trace amounts of genomic DNA, after which the DNase-treated RNA samples were quantified using a P300 Nanophotometer (Implen Inc., USA) and were stored at -80°C.

### Reverse transcription PCR

Reverse transcription (RT) was performed using SuperScript III reverse transcriptase (Life Technologies, Canada) with 500 ng of DNase-treated total RNA and 2 pmol of the gene-specific primer DRB674. Reactions were set up as per the manufacturer’s instructions and were incubated at 55°C for 1 hr. A negative control reaction in which no reverse transcriptase enzyme was added was included to verify the absence of genomic DNA in the RNA samples. PCR was performed using 2 μL of the cDNA template and the primer pairs DRB674-DRB253, DRB674-DRB254a and DRB674-DRB255. Amplification was conducted using Taq DNA polymerase (New England Biolabs, Canada) as per the manufacturer’s protocol except that the reactions included 5% v/v DMSO. The resulting PCR products were analyzed by electrophoresis using a 1% w/v agarose gel and 1× Tris Borate EDTA (TBE) buffer and were visualized by staining with ethidium bromide.

### Primer extension analysis

Primer extension was performed using a 6-carboxyfluorescein (FAM)—labeled primer, DRB674, as previously described [33] with modifications. Briefly, a 15 µL reaction containing 40 μg of DNase—treated RNA and 0.6 pmol of 5′-FAM-labeled primer was incubated at 65°C for 5 min and then chilled on ice. Next, 3 μL of SuperScript III reverse transcriptase (600U), 1.5 μL of RNaseOUT Recombinant Ribonuclease Inhibitor (Life Technologies, Canada), 3 μL of dNTPs (10 mM each), 1.5 μL of 0.1M dithiothreitol (DTT) and 6 μL of 5× First-Strand Buffer (Life Technologies, Canada) were added to the reaction, and the reaction was incubated at 55°C for 2 hr. An extra 1 μL of SuperScript III reverse transcriptase (200U) was added after 1 hr of incubation. Then, the reaction was heated at 70°C for 15 min, after which 1 μL (5U) of RNase H (New England Biolabs, Canada) was added and the reaction was incubated at 37°C for 30 min. This was followed by phenol/chloroform extraction and ethanol precipitation of the cDNA. The resulting cDNA pellet was air dried and then sent to TCAG for DNA sizing analysis. The primer extension analysis was performed twice in total.

### Electrophoretic mobility shift assay (EMSA)

The DNA probes used for EMSAs were amplified by PCR and were gel-purified using the Wizard SV Gel and PCR Clean-Up System (Promega, Canada). In addition, two pairs of long oligonucleotides (40 nt each), LC12—LC13 and LC14—LC15, were synthesized and used as probes in EMSAs. The complementary oligonucleotides LC12 and LC13 were used to generate probe 1 (P1) and contained the putative CfaR binding site, while the complementary pair LC14 and LC15 were used to generate the negative control probe (P2) and corresponded to the coding region of the *cfaR* gene. The oligonucleotide pairs were incubated at 95°C for 5 min and then slowly cooled to room temperature to allow annealing of the oligonucleotides. The DNA probes were either 3′ end-labeled using the Biotin 3′ End DNA Labeling Kit (Promega, Canada) or were unlabeled. The DNA-protein binding reactions were performed using the LightShift Chemiluminescent EMSA Kit (Fisher Scientific, Canada) according to the manufacturer’s instructions. Reactions containing unlabeled DNA and protein were analyzed by non-denaturing PAGE and the DNA was visualized afterwards using ethidium bromide. Reactions containing biotin-labeled probe and protein were analyzed by non-denaturing PAGE, after which the DNA was transferred to nitrocellulose membrane by contact blotting and then probed with anti-Biotin-alkaline phosphatase antibodies according to the manufacturer’s instructions (Fisher Scientific, Canada). Visualization of the DNA was then performed using the Chemiluminescent Nucleic Acid Detection Module (Fisher Scientific, Canada) and the ImageQuant LAS 4000 Digital Imaging System (GE Healthcare, Canada).

### Glutaraldehyde cross-linking of CfaR proteins

Crosslinking reactions consisted of purified CfaR protein (80 pmol) and 20 mM sodium phosphate buffer (pH 7.5) in a final volume of 20 μL. The reactions were initiated with the addition of 1 μL of a 2.3% w/v glutaraldehyde solution and were incubated for 5 minutes at 37°C. Termination of the reactions was achieved by the addition of 2 μL of a 1 M Tris-HCl solution (pH 8.0), after which the cross-linked proteins were separated by SDS-PAGE on a 12% polyacrylamide gel and were visualized by staining with Coomassie brilliant blue.

### Construction of *cfaR*, *orf1* and *cfaR+orf1* overexpression plasmids

Construction of the *cfaR* overexpression vector, pRLDR51-1, was described previously [19]. DNA fragments containing *orf1* alone and *cfaR* + *orf1* were amplified by PCR using Phusion DNA Polymerase according to the manufacturer’s instructions, except that DMSO (5% v/v final concentration) was included in the reactions. The resulting products were digested with XbaI (New England Biolabs, Canada) and were ligated into similarly digested pRLDB50-1a to generate the *orf1* and *cfaR* + *orf1* overexpression plasmids pRLDB81 and pRLDB891, respectively. The correct orientation of the inserts was confirmed by digestion with BamHI for pRLDB81 and with PstI, SmaI and NcoI for pRLDB891 (New England Biolabs, Canada), after which the constructed plasmids were sequenced to confirm the fidelity of the inserts. The expression plasmids were then introduced into *E*. *coli* ET12567/ pUZ8002 prior to transfer into *S*. *scabies* 87–22 by intergeneric conjugation [28].

### Extraction and analysis of CFA-L-Ile production

CFA-L-Ile was extracted from SFMB cultures of *S*. *scabies* and was quantified by analytical HPLC as described previously [16].

### Bioinformatics analysis

Identification of protein domains within the CfaR and ORF1 amino acid sequences was performed using the Pfam database (http://pfam.xfam.org/) [34]. The logo for the PAS-LuxR protein binding sites was generated using the WebLogo server (http://weblogo.berkeley.edu/logo.cgi) [35]. Amino acid sequence alignments of the PAS and LuxR domains from CfaR and other PAS-LuxR proteins in the database were generated using ClustalW within the Geneious version 6.1.2 software (Biomatters Ltd.). The accession numbers for the protein sequences used in the alignments are listed in S2 Table. Phylogenetic trees were constructed from the alignments using the maximum likelihood method in the MEGA 5.2.1 program [36]. Bootstrap analyses were performed with 1000 replicates in each algorithm.

## Results and Discussion

### CfaR binds to a single site located in the *cfa1* promoter region

**Fig 2:**
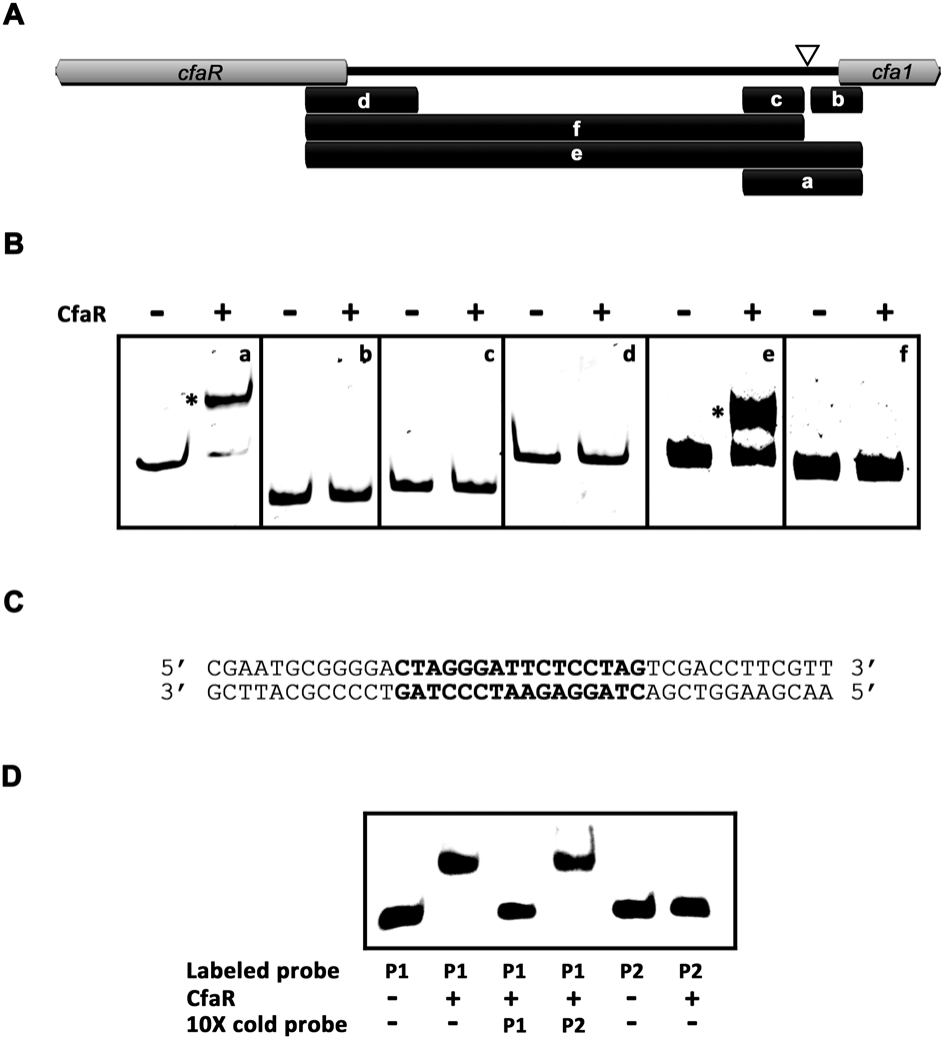
CfaR^full^-HIS_6_ binds to a single site within the *cfaR—cfa1* intergenic region. (A) Map of the *cfaR—cfa1* intergenic region showing the location of the DNA fragments (indicated by the black bars and labeled *a—f*) used for EMSAs. The position of the 16 bp palindrome identified upstream of *cfa1* is indicated with the white triangle. (B) EMSA s for CfaR^full^—HIS_6_ with the DNA fragments *a—f*. Reactions contained 50 ng of DNA with (result+) and without (-) CfaR^full^—HIS_6_ protein (3.7 pmol). DNA-protein complexes observed are indicated with *. (C) Sequence of the 40 bp oligonucleotide P1 probe used for EMSAs. The 16 bp palindromic sequence identified upstream of *cfa1* is shown in bold. (D) EMSA results for CfaR^full^—HIS_6_ with the P1 oligonucleotide probe. Reactions contained 0.1 pmol of biotin-labeled probe with (+) and without (-) CfaR^full^—HIS_6_ protein (2 pmol). Negative control reactions contained the 40 bp biotin-labeled oligonucleotide P2 probe in place of P1. In addition, competition assays were performed in which an excess (10×) of unlabelled (cold) probe (P1 or P2) was included in the reaction. DNA-protein complexes observed are indicated with *.

**Fig 3:**
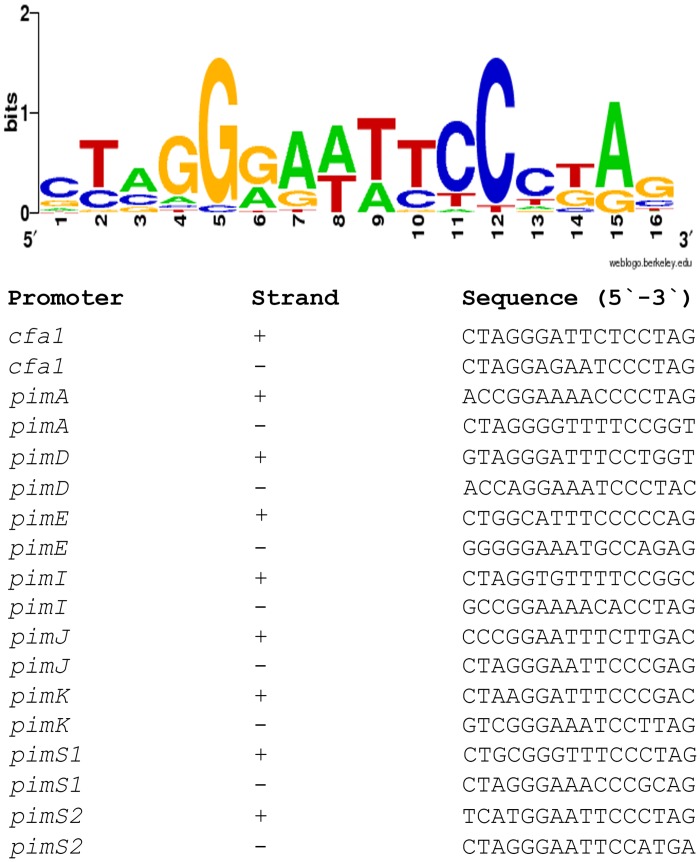
Sequence logo of PAS-LuxR protein binding sites. The logo was constructed using WebLogo [35] with the PimM and CfaR binding sites shown below. The overall height of the stack reflects the sequence conservation at that position, and the height of the letters within the stack designates the relative frequency of the corresponding base at that position [38].

Previous transcriptional studies showed that CfaR is required for expression of several coronafacoyl phytotoxin biosynthetic genes [19], and overexpression of CfaR has been demonstrated to enhance phytotoxin production [16]. Given that the biosynthetic genes are expressed as a large polycistronic transcript [19], it was hypothesized that CfaR may control gene activation from the promoter region upstream of *cfa1*, which is the first gene in the operon (Fig 1A). To investigate this further, CfaR was overexpressed and purified from *E*. *coli* as a C-terminal 6 × histidine tagged protein (CfaR^full^ –HIS_6_), after which it was used in EMSAs along with six DNA fragments covering different parts of the intergenic region between *cfaR* and *cfa1* (Fig 2A). As shown in Fig 2B, the CfaR^full^ –HIS_6_ could only bind to two of the DNA fragments (*a* and *e*), both of which covered a 264 bp region immediately upstream of the predicted *cfa1* start codon (Fig 2A). Within this region, a 16 bp imperfect palindromic DNA sequence was identified manually (positions -94 to -79 relative to the *cfa1* translation start codon; Fig 2A and 2C), and the sequence was found to be highly similar to the previously described PimM binding site consensus sequence CTVGGGAWWTCCCBAG (Fig 3) [23, 37]. EMSAs using DNA fragments lacking this palindrome confirmed that it is essential for binding of CfaR^full^ –HIS_6_ to DNA (Fig 2B). Furthermore, CfaR^full^ –HIS_6_ could readily bind to a 40 bp labeled oligonucleotide probe (P1) containing only the palindrome and some DNA flanking sequence (Fig 2C and 2D) whereas it did not bind to a control 40 bp probe (P2; Fig 2D) corresponding to the *cfaR* coding region (see Materials and Methods). Finally, binding to the labeled P1 probe was abolished when an excess of unlabeled P1, but not P2, was included in the reaction mixture (Fig 2D), indicating that the interaction between CfaR^full^ –HIS_6_ and P1 is highly specific.

**Fig 4:**
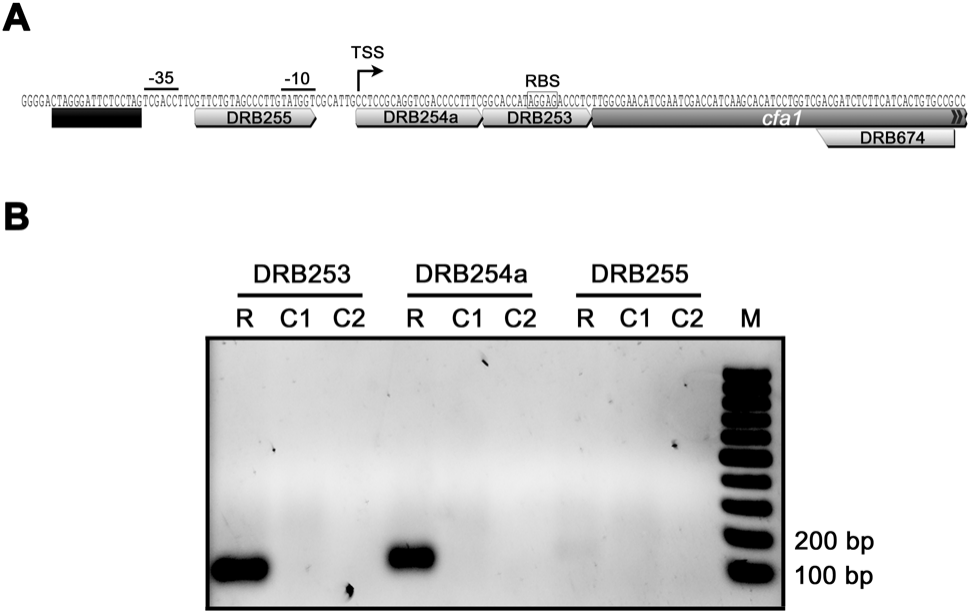
Mapping the transcription start site of *cfa1*. (A) Organization of the *cfa1* promoter region. The putative -10 and -35 hexanucleotide sequence boxes and the putative ribosome binding site (RBS) are shown along with the predicted CfaR binding site, which is indicated by the black bar. Also shown are the binding sites for the primers DRB253, DRB254a, DRB255 and DRB674, which were used for low resolution transcript mapping by RT-PCR. The transcription start site (TSS) as determined by non-radioactive primer extension analysis is also indicated. (B) Results of the low resolution transcription start site mapping by RT-PCR. Reverse transcription was performed using *S*. *scabies* Δ*txtA/*pRLDB51-1 total RNA and using the gene-specific primer DRB674. This was followed by PCR using the reverse primer DRB674 and the forward primers DRB253, DRB254a or DRB255. The resulting products were then analyzed by agarose gel electrophoresis. R, PCR reactions using cDNA as template; C1, control PCR reactions using RNA (without reverse transcription) as template; C2, control PCR reactions using water as template.

The location of the CfaR binding site within the *cfa1* promoter was further characterized by mapping the *cfa1* TSS. Total RNA was isolated from a *S*. *scabies* strain (Δ*txtA/*pRLDB51-1) that overexpresses the *cfaR* gene [19] and produces high levels of the coronafacoyl phytotoxins [16], and RT-PCR was performed using a single reverse primer and different forward primers (Fig 4A) in order to identify the approximate location of the TSS. As shown in Fig 4B, two of the forward primers (DRB253 and DRB254a) allowed for amplification of a PCR product from the cDNA template whereas the third forward primer (DRB255) did not, indicating that the TSS was most likely located somewhere between DRB254a and DRB255. This was verified using non-radioactive primer extension analysis, which identified a C residue located 40 bp upstream of the *cfa* translation start site as the TSS. A putative -10 box (TATGGT) and a -35 box (TCGACC) separated by 18 nt is situated upstream of the C residue (Fig 4A), and these features are consistent with the previously described consensus sequence (TTGACN— N_16-18_—TASVKT) for streptomycete *E*. *coli σ* ^70^-like promoters [39]. Interestingly, the palindromic sequence required for CfaR binding is located immediately upstream of the putative -35 box (Fig 4A), an arrangement that is similar to what has been described for promoters activated by PimM (the binding site of which typically overlaps the -35 box) [23]. Most likely, this arrangement allows for direct contact between the transcriptional activator and RNA polymerase in order to recruit RNA polymerase to the target promoter [40].

It is noteworthy that the CfaR^full^ –HIS_6_ protein did not bind to the DNA fragments *d* and *f*, which cover the promoter region for the *cfaR* gene (Fig 2A and 2B). This suggests that CfaR does not regulate its own expression, a finding that is consistent with previous transcriptional data from *S*. *scabies* [19] and is also consistent with the observation that PimM does not regulate its own expression [22]. In addition, the entire sequence of the coronafacoyl phytotoxin biosynthetic gene cluster was screened for other potential CfaR binding sites, and although a possible binding sequence was found within the *cfa6* gene, the CfaR^full^ –HIS_6_ protein did not bind to this site in EMSAs (data not shown). Therefore, it appears that CfaR regulates coronafacoyl phytotoxin production using a single DNA binding site within the entire gene cluster.

### The CfaR PAS domain is required for DNA binding and protein dimerization *in vitro*

**Fig 5:**
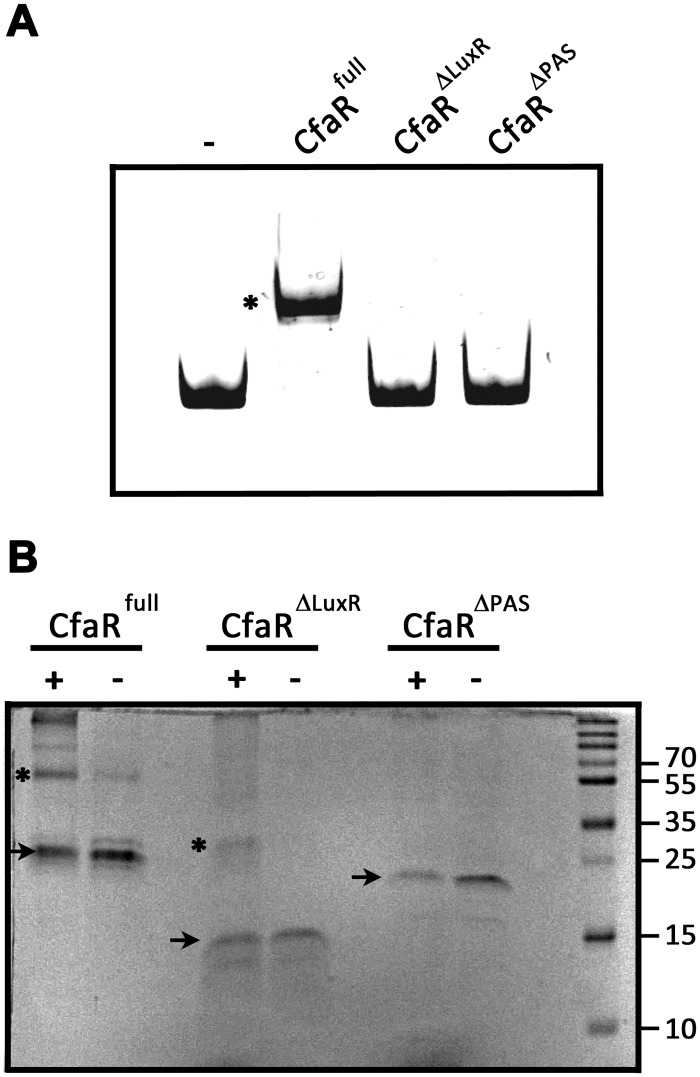
The CfaR PAS domain is required for DNA binding and protein dimerization. (A) EMSA results using CfaR^full^-HIS_6_, CfaR^ΔLuxR^-HIS_6_ and CfaR^ΔPAS^-HIS_6_ with DNA fragment *a* (Fig 2A). A control reaction (-) in which no protein was added was also included. The DNA-protein complex observed is indicated with *. (B) Analysis of protein dimerization using chemical crosslinking and SDS-PAGE. CfaR^full^-HIS_6_, CfaR^ΔLuxR^-HIS_6_ and CfaR^ΔPAS^-HIS_6_ were either treated with glutaraldehyde (+) or with solvent alone (-), after which the proteins were separated by SDS-PAGE and were visualized by staining with Coomassie brilliant blue. Protein monomers are indicated with black arrows and dimers with *. The sizes (in kDa) of the protein molecular weight marker (M) bands used for size estimation are also shown.

*In vitro* studies on the *S*. *natalensis* PimM protein have shown that the DNA binding activity of the protein requires the LuxR DNA binding domain but not the PAS domain, and that removal of the PAS domain actually enhances the DNA binding activity of the protein [23]. To investigate the role of the PAS and LuxR domains in the binding of CfaR to DNA, EMSAs were performed using two different truncated forms of the protein, CfaR^ΔLuxR^ –HIS_6_ and CfaR^ΔPAS^ –HIS_6_, which lack the LuxR and the PAS domain, respectively (Fig 1B). Fig 5A shows that while the CfaR^full^ –HIS_6_ protein could bind to the target DNA, neither of the truncated forms showed any DNA binding activity in the assay. This was expected in the case of CfaR^ΔLuxR^ –HIS_6_ since the protein lacks the HTH DNA binding motif; however, the lack of binding by CfaR^ΔPAS^ –HIS_6_ was surprising based on the previously described results for PimM. Given that transcriptional regulators that bind palindromic sequences normally do so as dimers, we next looked at whether deletion of the PAS domain affected the ability of CfaR to form homodimers using glutaraldehyde crosslinking and SDS-PAGE. As shown in Fig 5B, homodimeric forms of both CfaR^full^ –HIS_6_ (59.2 kDa) and CfaR^ΔLuxR^ –HIS_6_ (32.8 kDa) could be detected upon treatment with glutaraldehyde whereas only the monomeric form of CfaR^ΔPAS^ –HIS_6_ (20.0 kDa) could be observed under similar conditions, suggesting that the lack of DNA binding observed with CfaR^ΔPAS^ –HIS_6_ is most likely due to the inability of the protein to dimerize. It is noteworthy that a role for the CfaR PAS domain in protein dimerization is consistent with previous studies on the *Drosophila* circadian rhythm regulator (PER), the mouse aryl hydrocarbon receptor (AHR) and AHR nuclear translocator (ARNT), and the *Drosophila* single minded (SIM) transcription factor, where it was shown that the PAS domain in the respective proteins functions as a mediator of homo- and/or heterodimerization [41–44].

It is currently unclear as to why deletion of the PAS domain had such a drastically different effect on the DNA binding activity of CfaR as compared to PimM. Possibly, it is related to differences in the type and location of the fusion tag used for purifying each protein. In the case of PimM, the full length protein and its truncated versions were purified using an N-terminal GST tag [23], whereas a C-terminal HIS_6_ tag was used in the current study for purifying CfaR and its truncated versions. Given that GST fusion proteins have been reported to form dimers most likely due to GST-GST interactions [45–47], it is possible that the presence of the GST protein tag on the N-terminus of the PimM DNA binding domain allowed for dimerization of the protein in the absence of the PAS domain, thereby preserving the DNA binding activity of the truncated protein.

### Activation of coronafacoyl phytotoxin production by CfaR is enhanced by ORF1

**Fig 6:**
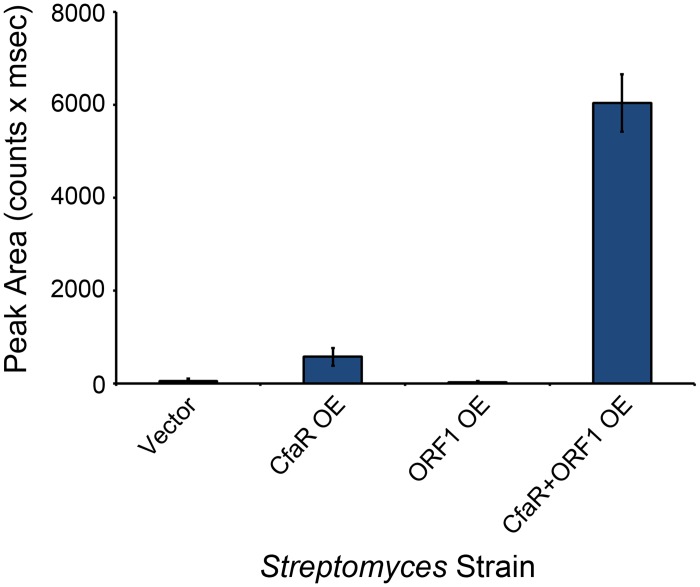
Coronafacoyl phytotoxin production is greatly enhanced by overexpression of both *cfaR* and *orf1*. *S*. *scabies* strains overexpressing *cfaR* alone, *orf1* alone or *cfaR* + *orf1* were cultured in SFMB medium for 7 days at 25°C, after which the culture supernatants were harvested and extracted with organic solvent. The resulting organic extracts were then analysed for CFA-L-Ile production by HPLC. Shown is the mean area of the CFA-L-Ile peak from six cultures of each strain, with error bars indicating the standard deviation. A *S*. *scabies* strain containing only the overexpression vector was used as a control in this experiment.

The *cfaR* gene has been shown to be co-transcribed with a downstream gene, *scab79581* (herein referred to as *orf1*) (Fig 1A), which encodes a protein of unknown function [19]. Given that co-transcribed genes are often involved in similar processes, we hypothesized that the ORF1 protein might also play a role in activating coronafacoyl phytotoxin production in *S*. *scabies*. To investigate this further, the *cfaR* and *orf1* genes were overexpressed individually and together in wild-type *S*. *scabies* 87–22, which normally produces undetectable or trace levels of CFA-L-Ile under laboratory conditions [16]. As shown in Fig 6, overexpression of *cfaR* significantly enhanced CFA-L-Ile production when compared to the vector control, a result that is consistent with previous *cfaR* overexpression studies in the Δ*txtA* thaxtomin A mutant background [16]. Interestingly, overexpression of *cfaR* + *orf1* led to an even greater increase (~10 fold) in CFA-L-Ile production when compared to overexpression of *cfaR* alone, while overexpression of *orf1* alone had no significant effect on CFA-L-Ile production when compared to the vector control (Fig 6). This suggests that ORF1 somehow augments the activation of CFA-L-Ile production by CfaR, though it is currently unclear as to how this would occur. Analysis of the ORF1 protein sequence using the Pfam database revealed the presence of a ThiF family domain (PF00899.16) and a nitroreductase domain (PF00881.19) situated at the N-terminus and central region of the protein, respectively. Interestingly, the ThiF family domain is found in enzymes such as the eukaryotic ubiquitin activating enzyme E1, the *E*. *coli* thiamine biosynthetic enzyme ThiF and the *E*. *coli* molybdenum cofactor biosynthetic enzyme MoeB, all of which are known to catalyze the adenylation of a target polypeptide at the C-terminal end [48]. In the case of ThiF and MoeB, the adenylated C-terminus of the target polypeptide is modified further to a thiocarboxylate, which then serves as a sulfur donor for cofactor biosynthesis [48]. Possibly, ORF1 is involved in some sort of post-translational modification of CfaR in order to enhance the ability of CfaR to elicit transcriptional activation. It is interesting to note that the co-transcription of genes encoding a PAS-LuxR homologue and an ORF1 homologue has not been found in other *Streptomyces* spp., suggesting that the regulation of coronafacoyl phytotoxin production may involve a novel mechanism.

### Phylogenetic analysis indicates that CfaR is a novel member of the PAS-LuxR protein family

**Fig 7:**
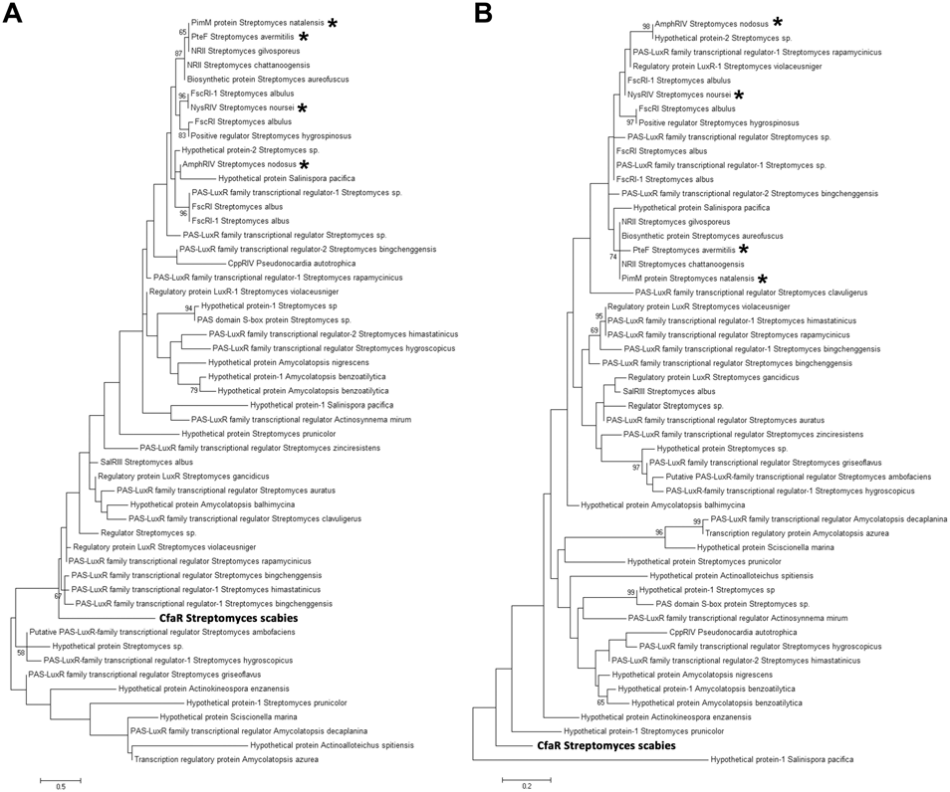
Phylogenetic analysis of PAS-LuxR family proteins from *Streptomyces* and other actinomycetes. The phylogenetic trees were constructed based on the amino acid sequences of the PAS domain (A) and the LuxR DNA binding domain (B). The trees were constructed using the maximum likelihood algorithm, and bootstrap values ≥50% for 1000 repetitions are shown. The scale bar indicates the number of amino acid substitutions per site. Family members that have been shown experimentally to be functionally interchangeable are indicated with *.

Previously, it was proposed that CfaR may represent a novel member of the PAS-LuxR protein family based on phylogenetic analysis using the complete amino acid sequence of CfaR and other PAS-LuxR proteins from the database [49]. This analysis demonstrated that CfaR forms a distinct lineage among the different PAS-LuxR family members, though it was unclear as to whether this is mainly due to sequence differences within the PAS domain and/or the LuxR domain, or due to differences within the inter-domain regions only. To examine this further, phylogenetic trees were constructed using only the amino acid sequences of the PAS domain (Fig 7A) or the LuxR DNA binding domain (Fig 7B) from CfaR and other PAS-LuxR family members. Interestingly, the resulting trees showed that both the PAS and LuxR domains from CfaR form a distinct clade among the corresponding domain sequences from other family members (Fig 7A and 7B), indicating that the uniqueness of the CfaR amino acid sequence can be extended into these conserved domains. It is also interesting to note that regardless of the sequence used to generate the tree (PAS domain, LuxR domain or full length protein), the phylogenetic analyses showed that CfaR is distantly related to the previously characterized PAS-LuxR family members PimM, PteF, AmphRIV and NysRIV (Fig 7A and 7B) [49], all of which are associated with polyene antifungal antibiotic biosynthetic gene clusters [37]. It has been shown that PteF, AmphRIV and NysRIV are able to complement a *S*. *natalensis* Δ*pimM* mutant [37] and that PimM is able to complement a *S*. *avermitis* Δ*pteF* mutant [24]. In addition, the purified PimM protein can bind to the predicted target sites for PteF, AmphRIV and NysRIV *in vitro* [37]. Together, these results imply that there is functional conservation among these members of the PAS-LuxR family of transcriptional regulators. Whether CfaR is also functionally interchangeable with PimM and similar PAS-LuxR proteins will require further investigation; however, the analyses performed here suggest that CfaR may be functionally distinct from these other family members.

## Concluding Remarks

This study has established that CfaR is a novel PAS-LuxR family protein that directly activates the expression of the *S*. *scabies* coronafacoyl phytotoxin biosynthetic genes by binding to a single site within the *cfa1* promoter region. This is the first report detailing the mechanism of coronafacoyl phytotoxin biosynthetic gene regulation by CfaR, and other than studies focused on PimM, it is the only other study to date that describes the biochemical characterization of a member of the PAS-LuxR protein family, which is highly represented among *Streptomyces* spp. and other actinomycetes. We also demonstrated that the PAS domain of CfaR is required for DNA binding and protein dimerization, a function that has not been previously described for this domain within the PAS-LuxR protein family. Given that PAS domains are known to function as sensory domains for controlling the regulatory activity of the corresponding protein [20], we suspect that the PAS domain in CfaR has the additional role of controlling the DNA binding activity of the protein in response to a ligand or some other stimulus, and we are currently examining this further. It has been shown that the coronafacoyl phytotoxin biosynthetic genes are expressed during colonization of plant tissues [19] and that production of CFA-L-Ile only occurs in media containing plant-derived components [16], and so it is possible that one or more plant-derived compounds serves as a signal for activating gene expression by CfaR. A particularly interesting finding from this study was the demonstration that the co-transcribed *orf1* gene is also involved in activating phytotoxin production, and we are currently investigating the precise role of the corresponding protein. Furthermore, the involvement of other regulators of *cfaR* via transcriptional or translational control is an area of interest given that a number of the *bld* (bald) gene global regulators that control morphological differentiation and secondary metabolism in *Streptomyces* spp. are known to modulate transcription of the *cfaR* gene or translation of *cfaR* mRNA in *S*. *scabies* [19, 50].

## Supporting Information

S1 Table Oligonucleotides used in this study. (DOCX)

S2 Table Accession numbers of PAS-LuxR protein sequences used for the phylogenetic analyses. (DOCX)
