# Supplementary material for: Regulation of Coronafacoyl Phytotoxin Production by the PAS-LuxR Family Regulator CfaR in the Common Scab Pathogen Streptomyces scabies
Source: PLoS One. 2015 Mar 31;10(3):e0122450. doi: 10.1371/journal.pone.0122450 (PMC4380410; doi:10.1371/journal.pone.0122450)
Supplement: S1 Table — (DOCX) [file pone.0122450.s001.docx]

**Table S1.** **Oligonucleotides used in this study.**

| **Primer** | **Sequence (5**′**🡪3**′**)** † | **Use** |
| --- | --- | --- |
| DRB252 | GCACAGGAAGGAGGAGAACC | Forward primer for amplification of EMSA probes *a* and *c* |
| DRB253 | GGCACCATAGGAGACCCTCT | Forward primer for *cfa1*  TSS mapping by RT-PCR |
| BRD254a | CCTCCGCAGGTCGACCCCTTTC | Forward primer for *cfa1*  TSS mapping by RT-PCR |
| DRB255 | GTTCTGTAGCCCTTGTATGG | Forward primer for *cfa1*  TSS mapping by RT-PCR |
| DRB283 | CGGCACAGTGATGAAGAGAT | Reverse primer for amplification of EMSA probes *a*, *b* and *e* |
| DRB674 | CGGCACAGTGATGAAGAGATCGTC | Reverse primer for *cfa1*  TSS mapping by RT-PCR and primer extension analysis |
| LC1 | GCG**CATATG**GCGAAATCAGGAGACCCGTC | Forward primer for construction of the overexpression plasmids pET30b/CfaR and pET30b/CfaR1.1 |
| LC3 | GCG**AAGCTT**TGCGCTCTCCAGCTCGGGGT | Reverse primer for construction of the overexpression plasmids pET30b/CfaR and pET30b/CfaR1.3 |
| LC4 | GCGC**TCTAGA**CTCTGTACCACCTGACGGAAG | Reverse primer for construction of the overexpression plasmid pRLDB891 |
| LC5 | GCGC**TCTAGA**TCCTGCCGGCCCTTCGGTAA | Forward primer for construction of the overexpression plasmids pRLDB81 and pRLDB891 |
| LC6 | GCGC**TCTAGA**CCGAACACGCTCATCCCGTC | Reverse primer for construction of the overexpression plasmid pRLDB81 |
| LC9 | GAACGCGTCGTTCGCCTCCA | Forward primer for amplification of EMSA probes *d, e* and *f* |
| LC10 | GCACGACCGGCTTTCCCACA | Reverse primer for amplification of EMSA probe *d* |
| LC12 | CGAATGCGGGGACTAGGGATTCTCCTAGTCGACCTTCGTT | Oligonucleotide EMSA probe containing the CfaR binding site; complimentary with LC13 |
| LC13 | AACGAAGGTCGACTAGGAGAATCCCTAGTCCCCGCATTCG | Oligonucleotide EMSA probe containing the CfaR binding site; complimentary with LC12 |
| LC14 | TGTGGTTCGCGTCCATGGGTGTCAGGCGGATGCTTAAGTC | Negative control oligonucleotide EMSA probe; complimentary with LC15 |
| LC15 | GACTTAAGCATCCGCCTGACACCCATGGACGCGAACCACA | Negative control oligonucleotide EMSA probe; complimentary with LC14 |
| LC17 | GCGC**CATATG**CACACGGACAGGGACTGCCT | Forward primer for construction of the overexpression plasmid pET30b/CfaR1.3 |
| LC20 | CCCGCATTCGGGTTGGCGTC | Reverse primer for amplification of EMSA probes *c* and *f* |
| LC21 | GACCTTCGTTCTGTAGCCCT | Forward primer for amplification of EMSA probe *b* |
| LC45-1 | GCGC**AAGCTT**CACCTCGGCGAGTGTCGTGA | Reverse primer for construction of the overexpression plasmid pET30b/CfaR1.1 |

† Non-homologous extensions are underlined, while engineered restriction sites are indicated in bold.
