# Supplementary material for: Regulation of Coronafacoyl Phytotoxin Production by the PAS-LuxR Family Regulator CfaR in the Common Scab Pathogen Streptomyces scabies
Source: PLoS One. 2015 Mar 31;10(3):e0122450. doi: 10.1371/journal.pone.0122450 (PMC4380410; doi:10.1371/journal.pone.0122450)
Supplement: S2 Table — (DOCX) [file pone.0122450.s002.docx]

**Table S2. Accession numbers of PAS-LuxR homologues used for phylogenetic trees.**

| **Name** | **Accession number** |
| --- | --- |
| AmphRIV_Streptomyces nodosus | AAV37062.1 |
| Biosynthetic protein_Streptomyces aureofuscus | ACD75765.1 |
| CfaR_Streptomyces scabiei | YP_003493449.1 |
| CppRIV_Pseudonocardia autotrophica | ABV83236.1 |
| FscRI-1_Streptomyces albulus | WP_016577550.1 |
| FscRI-1_Streptomyces albus | WP_003946517.1 |
| FscRI_Streptomyces albulus | WP_016570421.1 |
| FscRI_Streptomyces albus | YP_007749177.1 |
| Hypothetical protein-1_Amycolatopsis benzoatilytica | WP_020659271.1 |
| Hypothetical protein-1_Salinispora pacifica | WP_018725106.1 |
| Hypothetical protein-1_Streptomyces prunicolor | WP_019062029.1 |
| Hypothetical protein-1_Streptomyces sp | WP_018554963.1 |
| Hypothetical protein-2_Streptomyces sp. | WP_017946556.1 |
| Hypothetical protein_Actinoalloteichus spitiensis | WP_016700531.1 |
| Hypothetical protein_Actinokineospora enzanensis | WP_018680905.1 |
| Hypothetical protein_Amycolatopsis balhimycina | WP_020638021.1 |
| Hypothetical protein_Amycolatopsis benzoatilytica | WP_020659273.1 |
| Hypothetical protein_Amycolatopsis nigrescens | WP_020673261.1 |
| Hypothetical protein_Salinispora pacifica | WP_018827366.1 |
| Hypothetical protein_Sciscionella marina | WP_020494548.1 |
| Hypothetical protein_Streptomyces prunicolor | WP_019062028.1 |
| Hypothetical protein_Streptomyces sp. | WP_019328950.1 |
| NRII_Streptomyces chattanoogensis | ACM45445.1 |
| NRII_Streptomyces gilvosporeus | ACM45446.1 |
| NysRIV_Streptomyces noursei | AAF71781.1 |
| PAS domain S-box protein_Streptomyces sp. | WP_016467294.1 |
| PAS-LuxR family transcriptional regulator-1 _Streptomyces bingchenggensis | YP_004958910.1 |
| PAS-LuxR family transcriptional regulator-1_Streptomyces himastatinicus | WP_009712487.1 |
| PAS-LuxR family transcriptional regulator-1_Streptomyces rapamycinicus | WP_020866044.1 |
| PAS-LuxR family transcriptional regulator-1_Streptomyces sp. | WP_018469600.1 |
| PAS-LuxR family transcriptional regulator-2_Streptomyces bingchenggensis | YP_004967281.1 |
| PAS-LuxR family transcriptional regulator-2_Streptomyces himastatinicus | WP_009712640.1 |
| PAS-LuxR-family transcriptional regulator-1_Streptomyces hygroscopicus | YP_006242418.1 |
| PAS-LuxR family transcriptional regulator_Actinosynnema mirum | YP_003102220.1 |
| PAS-LuxR family transcriptional regulator_Amycolatopsis decaplanina | WP_007029767.1 |
| PAS-LuxR family transcriptional regulator_Streptomyces auratus | WP_006607812.1 |
| PAS-LuxR family transcriptional regulator_Streptomyces avermitilis | NP_821584.1 |
| PAS-LuxR family transcriptional regulator_Streptomyces bingchenggensis | YP_004967325.1 |
| PAS-LuxR family transcriptional regulator_Streptomyces clavuligerus | WP_003959350.1 |
| PAS-LuxR family transcriptional regulator_Streptomyces griseoflavus | WP_004936272.1 |
| PAS-LuxR family transcriptional regulator_Streptomyces hygroscopicus | YP_006242550.1 |
| PAS-LuxR family transcriptional regulator_Streptomyces rapamycinicus | WP_020866268.1 |
| PAS-LuxR family transcriptional regulator_Streptomyces sp. | WP_019328946.1 |
| PAS-LuxR family transcriptional regulator_Streptomyces zinciresistens | WP_007491099.1 |
| PimM protein_Streptomyces natalensis | CAM35468.1 |
| Positive regulator_Streptomyces hygrospinosus | AFQ68276.1 |
| Putative PAS-LuxR-family transcriptional regulator_Streptomyces ambofaciens | CAJ89366.1 |
| Regulator_Streptomyces sp. | ABB88526.1 |
| Regulatory protein LuxR-1_Streptomyces violaceusniger | YP_004814000.1 |
| Regulatory protein LuxR_Streptomyces gancidicus | WP_006129792.1 |
| Regulatory protein LuxR_Streptomyces violaceusniger | YP_004813791.1 |
| SalRIII_Streptomyces albus | ABG02265.1 |
| Transcription regulatory protein_Amycolatopsis azurea | WP_005165576.1 |
